# Y-Site Compatibility Studies of Parenteral Nutrition and Other Intravenous Medications in Neonatal and Pediatric Patients: A Review of the Literature Evidence

**DOI:** 10.3390/pharmaceutics16020264

**Published:** 2024-02-12

**Authors:** Aleksandra Gostyńska, Tomasz Przybylski, Magdalena Ogrodowczyk

**Affiliations:** Department of Pharmaceutical Chemistry, Poznan University of Medical Sciences, Rokietnicka 3, 60-806 Poznan, Poland; tomasz.przybylski@student.ump.edu.pl

**Keywords:** parenteral nutrition, intravenous drugs, compatibility, pediatric patients

## Abstract

Background: Polytherapy in neonatal and pediatric patients requiring parenteral nutrition (PN) administration is a challenging task. Due to limited intravenous access, the Y-site administration of medication with PN admixtures is sometimes inevitable. Aim: This review aims to summarize the evidence on the compatibility of the Y-site of intravenous medications and PN admixtures in neonatal and pediatric settings. Methods: A literature review of the PubMed database was conducted. Articles published between January 1995 and November 2023 concerning the compatibility of intravenous medications in pediatric-dose PN admixtures or with intravenous lipid emulsions only were included. Studies concerning the compatibility/stability of the ingredients of PN admixtures and those concerning unapproved medications were excluded. Based on the methodology used, the quality of the research was assessed. Results: A total of fifteen studies were explored. Among fifty-five different drug substances assessed in the research reviewed, 56% (31/55) were found to be compatible, 13% (7/55) were assigned as incompatible, and for 31% (17/55), the data were ambiguous. None of the studies demonstrated an “A” grade (very high quality), and the grades “B”, “C”, and “D” were assigned to four, six, and five studies, respectively. The compatibility data are presented in two tables, the first concerning the simultaneous administration of medications with 2-in-1 PN formulations (without lipids) and the second, with 3-in-1 formulations (with lipids) and lipid emulsions. Conclusions: This review presents data on compatibilities between intravenously administered medications and PN mixtures intended for neonates and pediatric patients found in the PubMed database. It should be highlighted, however, that this work has some limitations. The clinical decisions on the simultaneous administration of intravenous medication with PN admixtures should be based not only on this review (including assessment of the quality of evidence) but also on manufacturer data, available electronic databases, and incompatibility data for PN admixtures dedicated to adult patients.

## 1. Introduction

Parenteral nutrition (PN) admixtures should be tailored to meet the specific nutritional needs of the child based on their age, weight, diagnosis, and growth requirements [1]. The complexity of PN admixture and the heightened risk of associated medication errors causing significant patient harm in acute care settings has resulted in this practice being classified as a high-alert medication by the Institute for Safety Medications Practices [2]. There are three main methods of conducting nutritional therapy in pediatric patients. Firstly, PN can be administered in the form of authorized pharmaceutical specialties provided as multi-chamber bags. These products are in line with current standards and meet the needs of a large group of preterm neonates, infants, young children, and adolescents [3,4]. Secondly, PN may be administered in the form of in-house compounded 3-in-1 formulations (containing amino acids, carbohydrates, and lipids as a source of macronutrients), either standardized or individualized, and prepared by hospital pharmacists. Thirdly, administration of a 2-in-1 PN solution (containing amino acids and carbohydrates as a source of macronutrients) and, concomitantly, intravenous lipid emulsion using two separate infusion lines may be conducted. Such an approach is recommended, especially for neonates and young children, due to the lower risk of lipid emulsion destabilization by the other components of the PN solution [5]. 

Pediatric patients often require the administration of other intravenous medications along with PN administration. Due to limited intravenous access, the use of Y-site administration in this group of patients is often inevitable. There are still gaps in the data regarding the compatibility of medications used in pediatric wards, which may concern up to 15% of the combinations used [6]. Potential complications of co-administration of incompatible medications include precipitation in infusion lines or central venous catheters, leading to infusion line occlusion. Administration of precipitate and large lipid droplets into the venous system can cause capillary embolization and local or systemic inflammatory responses, leading to venous thrombosis, chronic venous insufficiency, and even pulmonary embolism. Central venous catheter occlusion is the most common complication, occurring in up to 25% of patients. This situation can lead to a loss of ability to administer medications and PN through obstructed venous access [7,8]. The consequences of administering infusions with inadequate quality (the presence of precipitate) can be very serious for patients’ health and result in similar detrimental outcomes to administering lipid particles of considerable size, such as capillary embolization [9].

Administering two medications using one intravenous line requires special consideration and detailed analysis of the drug concentrations, infusion rates, diluents used for reconstitution, and proportions between components in the PN. Only such an approach can ensure the safety of therapy in such a special group of patients. This review aims to summarize the evidence on compatibility of the Y-site of intravenous medications and PN admixtures used in neonatal and pediatric settings. We also identify the methodology used to evaluate the compatibility and discuss the limitations of data extrapolation. 

## 2. Methods

### 2.1. Research Methodology 

**Figure 1:**
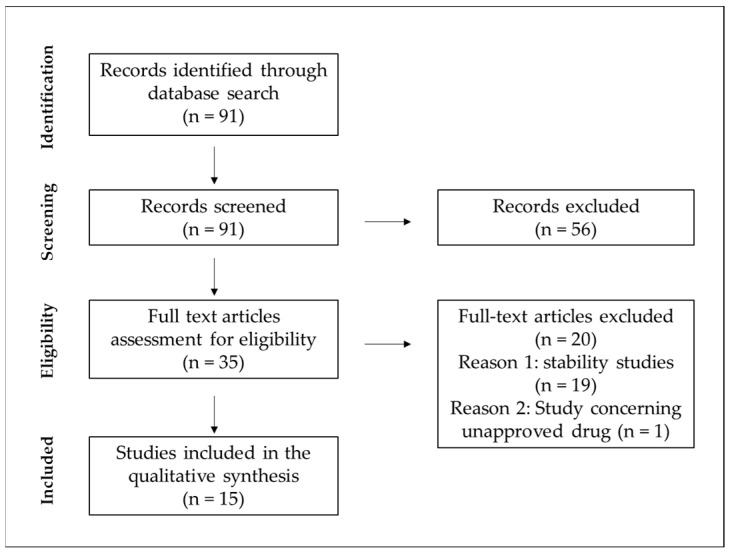
Flow diagram of the search methodology.

The research methodology of this study involved searching data based on six different configurations of three-keyword-based sets. The keyword sequence used to search the database was the following: “parenteral nutrition” and “compatibility” or “Y-site” and “neonatal” or “neonates” or “pediatric” or “paediatric”. Figure 1 presents the flow diagram of the search methodology. 

One electronic database, PubMed, was searched by two independent researchers, where a total of 91 results were found from January 1995 up to November 2023. As the exclusion criteria, we adopted the studies concerning the compatibility/stability of drugs being added to PN admixtures but not administered simultaneously using the Y-site. The second criterion was studies investigating the Y-site compatibility of PN admixtures with unapproved medications. After removing reviews, clinical trials, case reports, and articles not written in English, the resulting database was analyzed by abstract screening for the exclusion criteria. Following their application, 16 records were obtained on the compatibility of intravenous medications in neonatal and pediatric doses with 3-in-1 PN, 2-in-1 PN, or intravenous lipid emulsions. 

### 2.2. Research Quality Assessment

Inspired by the Stabilis 4.0 database (www.stabilis.org; accessed on 5 January 2024) grading system (that mainly concerns the quality of chemical stability research), for a better overview of the quality of the presented data, we developed a grading system for the assessment of the physicochemical compatibility of intravenous drugs with PN admixtures. This review used the following letter scale (A, B, C, or D) which scored the level of evidence and quality of the studies presented. 

The A grade was assigned to studies presenting a very high evidence level. These studies evaluated both the chemical stability of the drug substance using the HPLC method, which allowed for the effective separation of the drug and its degradation products, and the physical compatibility using instrumental methods. The physical compatibility was assessed, in the case of lipid-containing formulations (3-in-1 PN emulsions or lipid emulsions), using two instrumental methods recommended by the United States Pharmacopeia in Chapter <729> (USP <729>): (i) dynamic light scattering or classical light scattering methods for determination of the mean droplet diameter (MDD) and (ii) measurement of the percentage of fat residing in globules larger than 5 µm (PFAT5) by light obscuration or light extinction methods or, in the case of lipid-free formulations (2-in-1 PN solutions), instrumental methods for both counting sub-visual particles and turbidity assessment.

The B grade was assigned to studies that presented a high level of evidence. These studies did not necessarily evaluate the chemical stability of the drug substance; however, a physical compatibility assessment was performed using instrumental methods including, in the case of lipid-containing formulations (3-in-1 PN emulsions or lipid emulsions), two methods recommended by the USP <729>: MDD evaluation using dynamic light scattering or classical light scattering methods and measurement of the PFAT5 by light obscuration or light extinction methods or, in the case of lipid-free formulations (2-in-1 PN solutions), instrumental methods for both counting sub-visual particles and turbidity assessment. 

The C grade was assigned to studies that presented a medium level of evidence. These studies did not necessarily evaluate the chemical stability of the drug substance; however, a physical compatibility assessment was performed using instrumental methods, including, in the case of lipid-containing formulations (3-in-1 PN emulsions or lipid emulsions), at least one method recommended by the USP <729>: MDD evaluation using dynamic light scattering or classical light scattering methods or measurement of the PFAT5 by light obscuration or light extinction methods or, in the case of lipid-free formulations (2-in-1 PN solutions), at least one instrumental method for counting sub-visual particles or turbidity assessment. 

The D grade was assigned to studies that presented a low level of evidence. These studies only evaluated chemical stability or physical compatibility, but the methodology used was not comprehensive.

## 3. Results and Discussion

### 3.1. Compatibility Data 

The literature review identified 15 research works on the Y-site compatibility of PN and other intravenous medications in neonatal and pediatric patients [10,11,12,13,14,15,16,17,18,19,20,21,22,23,24]. We categorized them into two groups: those concerning a 2-in-1 PN solution, where the intravenous lipid emulsion was administered separately (Table 1), and those concerning compatibility studies of intravenous medications with 3-in-1 formulations either compounded by hospital pharmacists or authorized pharmaceutical specialties (Table 2). One study concerning the compatibility of intravenous medications with only an intravenous lipid emulsion was added to Table 2 [22]. A total of fifty-five different drug substances in concentrations used in neonatal and pediatric patients were explored. A total of 56% (31/55) were found to be compatible, 13% (7/55) were assigned as incompatible, and for 31% (17/55), the data were ambiguous. The result was considered ambiguous if the authors of the study included it in such a category or if several authors tested the same medication and obtained different results. The ambiguous data between authors was found for four drug solutions: aminophylline [14,17], ampicillin sodium [10,17,21,22,24], ceftazidime [17,21,24], and fosphenytoin sodium [13,21,24]. In the cases of acetazolamide sodium [17], acyclovir sodium [17], amiodarone [13], chlorothiazide sodium [17], pentobarbital sodium [13], phenobarbital sodium [13], and rifampicin [13], the results of the evaluated works indicated that concomitant administration of such medications with PN is contraindicated and can lead to therapy failure or serious consequences for patients’ health or life. 

### 3.2. Research Quality Evaluation

To determine the quality of the research, the method of determining the proportions between medications and PN admixtures, the sampling period, and the number of applied methods used for incompatibility identification were evaluated. Since the administration rates of both medications are crucial to properly evaluating the incompatibilities that may occur in clinical settings, this is an important parameter that should be considered when planning Y-site compatibility studies. To simulate clinical conditions, the proportion of medications coexisting in the infusion line should be determined concerning the extreme infusion rates of drug solutions being mixed. Such an approach was applied only by 40% (6/15) of the researchers [14,18,19,20,21,24]. Other researchers used the methodology introduced by the authors of the first works dealing with this topic, where only a 1:1 volume ratio was evaluated [25]. There are two known methods used for the assessment of compatibilities. The first is the static method, which is based on combining medications in tested ratios in a test tube. Such a method was used in all presented studies. The second is a dynamic method that involves sampling the combined medications infused by automatic pumps at a given rate and simulating their administration. Some authors preferred this method since it reflects clinical conditions and takes into account the possible influence of the infusion line material on the observed interactions [26,27]. There are also differences between authors in the sampling period. The most often used sampling periods were at the points 0 h and 4 h (60% (9/15) of analyzed works) [10,16,17,18,19,20,21,23,24]. However, 33% (5/15) of researchers collected their samples at more than four time points [12,13,15,17,23]. Nevertheless, the most important element in assessing the quality of conducted research evaluating a drug’s compatibility is not the sampling period but the type of methods used. To assess the quality of the studies presented in this review, we developed a four-grade letter scale (see Section 2) and assigned each study the appropriate letters: A, B, C, or D. None of the studies was assigned the A grade. Only four research groups applied five or more analytical methods to evaluate the compatibility between medications and PN admixtures and included both methods recommended by the USP for assessing injectable lipid emulsions, getting a B grade [19,20,21,24]. Those studies present a comprehensive approach and can be perceived as high-quality; however, since they are not assessing the chemical stability of the drugs, we could not assign them an A grade. Six studies were rated as being of medium quality (C grade) [10,12,13,18,22,23]. The remaining ones were based on selected methods and assigned with the D grade [11,14,15,16,17]. In some cases, the determination of particulate matter was made without the support of instrumental methods [14,15,16,17], which does not guarantee the detection of potential incompatibilities and indicates a poor quality of the research.

### 3.3. Compatibility Evaluation Methods

Determining the incompatibilities between intravenous medications and PN admixtures, either in a solution or an emulsion, is an important issue in ensuring safe therapy. The concomitant administration can be performed using a Y-site connector which is located in the lower part of the infusion line just before the distal end. Due to the short contact time of two medications administered in such a manner, which is less than 2 min, the chemical instability is considered at a low risk. Thus, it was rarely investigated (2 of the 15 assessed works) [11,23]. The HPLC method is much more often used in the stability assessment of PN admixture additives, e.g., vitamins [28,29]. Nevertheless, co-administration of medications with PN admixtures can affect the stability of the lipid emulsion or lead to changes in the drug substance’s ionic form [30]. The most common signs of drug–PN incompatibility are lipid emulsion breakdown manifested by pH or color changes and lipid emulsion phase separation, including creaming or coalescence processes [7,8,9]. Another important issue is the formation of a precipitate of active substances or excipients present in the drug dosage form. The combined administration of incompatible medications may also result in legal consequences for medical personnel and high costs of treatment of complications. Therefore, to ensure safe therapy, physicochemical compatibility is determined. A detailed analysis of the techniques used and the equipment applied by the scientists in the analyzed works is presented in Table 3 and Table 4. 

The analysis of studies whose authors undertook the problem of compatibility assessment between the intravenous medications and PN in the form of a solution allowed for distinguishing six scopes of interest: (i) the assessment of visible precipitation, color change, or gas production, (ii) analysis of the Tyndall beam effect, (iii) sub-visual particle counting, (iv) turbidity and (v) pH measurement, and (vi)drug concentration evaluation (Table 3). In the case of PN admixtures in the form of lipid emulsion, authors analyzed the appearance of visible precipitation, color change, and gas production, determined the particle size (MDD and PFAT5), and evaluated the changes in pH, osmolality, zeta potential, or drug concentration (Table 4). In the research methodology used to evaluate the compatibility of the PN and other intravenous drugs, the methods dedicated to PN admixtures both in the form of solution (2-in-1) and in the form of lipid emulsion (3-in-1), those dedicated uniquely to PN in the form of solution (2-in-1), and those dedicated uniquely to PN containing lipid emulsion (3-in-1) could be distinguished. Tests used for all types of PN included visual examination, pH, and drug concentration change evaluation. Additionally, for 2-in-1 PN admixtures uniquely, turbidity determination was preferred, and in the case of PN containing lipid emulsion zeta potential, osmolality and lipid emulsion particle size analyses were performed.

### 3.4. Limitations of Data Extrapolation

**Figure 2:**
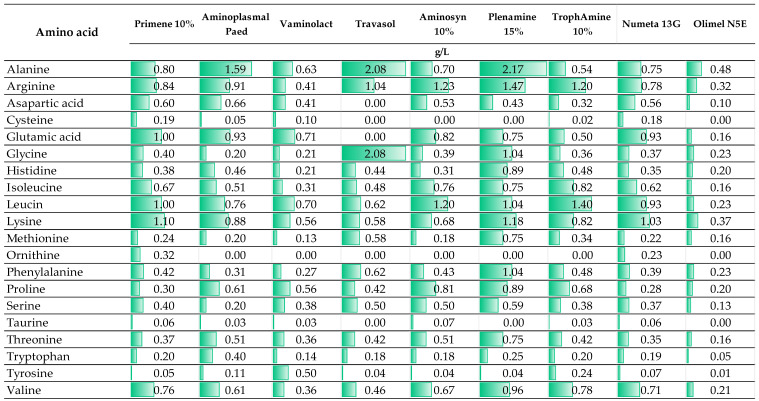
The composition of intravenous amino acid solutions for the preparation of neonatal or pediatric PN (The presented data are based on the manufacturer information (SmPC)).

The special nutritional requirements of newborns and children who differ from adults have necessitated the development of PN ingredients dedicated to this special group of patients. Generally, infants and young children have higher protein requirements per unit of body weight compared to adults due to the period of rapid growth and tissue development in their bodies. The adequate supply of essential amino acids, including histidine, isoleucine, leucine, lysine, methionine, phenylalanine, threonine, tryptophan, and valine, play a crucial role in their growth and development, and, as such, should be provided in proper amounts within PN. Moreover, some children and infants, especially preterm infants, due to illness or immaturity (this applies to premature infants), are unable to sufficiently, endogenously synthesize some essential amino acids, which in this condition become essential (conditionally essential). This concerns cysteine, tyrosine, glycine, arginine, proline, asparagine, and glutamine [31]. Nevertheless, the conditional indispensability of some of these amino acids is discussed [32,33]. Currently, few pediatric formulations of amino acid solutions are available on the market. In Figure 2, a comparison of the amino acid compositions of different products and their indications is provided. 

Interestingly, most amino acid solutions and commercially available PN admixtures in multi-chamber bags used in adult patients are registered from 2 years old. An example of such a preparation is Olimel N5E used by Staven et al. [24], in whose study the compatibility of intravenous medications was evaluated with two commercially available PN admixtures intended for central administration dedicated to neonates to 2 years (Numeta G16E) and children > 2 years (Olimel N5E). As shown in Figure 2, the quantitative composition of amino acids in Olimel N5E differed significantly from other presented preparations which are dedicated to patients from 1 day of life. As indicated in several works presented in this review, a change in qualitative and quantitative composition may determine a compatibility change [10,21,22]. This issue has not been well studied so far for amino acid solutions; however, it is already known that the stability of PN formulation depends on the amino acid solution used [34]; therefore, we cannot exclude the possibility that it does not affect compatibility with other medications. Other important components of compounded PN admixtures are glucose and electrolytes. These ingredients are provided mainly from a single preparation so their concentration in the final medication can be easily controlled. However, the role of phosphates (inorganic or organic) on the stability of PN admixture and therefore their impact on compatibility cannot be ignored. It is well known that PN formulations containing the organic source of phosphates are less prone to destabilization [35,36]. Therefore, drugs compatible with PN admixtures based on organic phosphates cannot be expected to behave in the same way when the phosphate source in PN admixtures is changed to inorganic. The analyzed works include research on compatibility with PN admixtures containing inorganic [10,11,12,13,14,15,16,17,22,23] and organic [18,19,20,21,24] phosphates.

On the contrary, vitamins and trace elements are ingredients provided in a complex pharmaceutical formulation, providing a panel of active ingredients. Several preparations of vitamins and trace elements indicated for pediatric patients are available on the pharmaceutical market, including Infuvite Pediatric, Peditrace, Soluvit N Infant, and Vitlipid N Infant. Scientists agree that the results obtained for a specific PN composition should not be extrapolated, especially when the obtained results would be transferred to different preparations of intravenous medications or PN formulations containing a different pharmaceutical preparation (e.g., another amino acid preparation) or higher concentrations of individual ingredients (e.g., electrolytes) [37,38,39,40].

**Figure 3:**
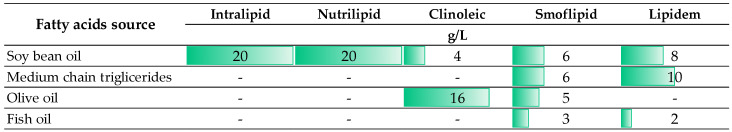
The oil phase compositions of intravenous lipid emulsions used for the preparation of neonatal or pediatric PN (The presented data are based on the manufacturer information (SmPC)).

Since there are multiple ways of providing PN therapy, in the literature concerning Y-site compatibility, PN is defined in various ways, either as 2-in-1 PN in the form of solution or as 3-in-1 PN admixture in the form of lipid emulsion. Lipid emulsion is a critical component of PN. It is prone to many destabilization factors. Due to their ability to neutralize repellent electrostatic interactions between lipid droplets, exposure to acidic conditions, especially at pH below 5.5, and high concentrations of calcium and magnesium ions are the main known destabilizers of lipid emulsions [41]. Infusing unstable lipid emulsions separately or as an ingredient of a PN with droplets that exceed the internal diameter of the pulmonary microvasculature increases the risk of embolic syndrome [42]. As shown in several studies, the type of lipid emulsion can affect compatibility [43,44,45]. This is a result of different oil compositions between preparations and, in some cases, various types or concentrations of excipients. A comparison of intravenous lipid emulsions used to prepare neonatal or pediatric PN is shown in Figure 3. 

Those differences are the reason why the data obtained by the authors for PN admixtures prepared based on one type of intravenous lipid emulsion should not be extrapolated to PN admixtures prepared using other preparations. Extrapolation of compatibility results should be avoided. Nevertheless, for safety reasons, it is advisable and necessary to extrapolate the results regarding incompatibility, regardless of whether the incompatibility was demonstrated for preparations intended for adults or children. It is also advisable to check the compatibility data not only in the available literature but also in electronic databases, e.g., Micromedex, Lexicomp, Medscape, or Drugs.com [46].

These data extrapolation limitations point to the need for continued research to improve infusion therapy safety in neonates and children requiring parenteral nutrition administration. 

## 4. Limitations

The presented review study has some limitations. Firstly, we searched only the PubMed database, omitting ScienceDirect, Scopus, Web of Science, and Google Scholar, and we did not present the compatibility data provided by the producers in the SmPC, making it possible that we overlooked some important incompatibility data. We justify this lack by the fact that the data from the manufacturer would have been difficult to assess in terms of the quality of the study because none of the PN manufacturers provided their methodology based on which they determined the incompatibility of individual drugs with PN. In this study, we decided to analyze only data from scientific research publications with a known methodology. Secondly, we did not analyze electronic databases, e.g., Micromedex, Lexicomp, Medscape, or Drugs.com. Moreover, data available in the scientific literature present data obtained on request, which also limits the clinical relevance of our study. Thirdly, the analysis of the source of financing of the studies showed that two of the presented studies were financed by the producer of the medications being studied [16,17], which could have affected the published results and suggests a bias in their data analyses. The other reviewed studies had no financing support [10,11,14,15,22,23] or were funded by regional or national institutions [12,13,18,19,20,21,24]. Nevertheless, we believe that the presented manuscript will help in determining incompatibilities between drugs and PN admixtures dedicated to neonatal and pediatric patients, as well as the appropriate research methodology and areas (medications) for which there is a need for further research.

## 5. Conclusions

This review presents data on compatibilities between intravenously administered medications and PN mixtures for neonates and pediatric patients found in the PubMed database. Research concerning compatibility studies was characterized by various evidence quality assessments, and none of the studies gained an A grade (very high quality). Among a total of fifty-five different drug substances assessed in the research reviewed, 56% (31/55) were found to be compatible, 13% (7/55) were assigned as incompatible, and for 31% (17/55), the data were ambiguous. It should be highlighted, however, that this work has some limitations. The clinical decisions on the simultaneous administration of intravenous medication with PN admixtures should be based not only on this review (including assessment of the evidence quality) but also on the manufacturer data, available electronic databases, and incompatibility data known for PN admixtures dedicated to adult patients.

## Figures and Tables

**Table 1:** Compatibility studies of intravenous medications and 2-in-1 PN solutions (without lipids).

Parenteral Nutrition	Drug Solution	Final Concentration of Drug	Diluent	Mixing Ratio of Drug + PN	Compatibility/Incompatibility (C/I) *	Ref./Grade (A,B,C,D) **
Two different PN solutions containing the following: Amino acids (TrophAmine, Plenamine) 38.2–58.3 g/L; Cysteine 0–1.5 g/L; Glucose 165–250 g/L; Sodium chloride 26.3–43.7 mmol/L; Sodium acetate 0–19.7 mmol/L; Sodium phosphate 13.2–17.5 mmol/L; Potassium chloride 19.7–14.6 mmol/L; Potassium acetate 13.15–14.6 mmol/L; Calcium gluconate 11.7–16.4 mmol/L; Magnesium sulfate 3.3–4.4 mmol/L; Multivitamin 16.7–21.9 mL/L; Zinc 5263–1167 µg/L; Copper 263–1666 µg/L; Selenium 22–200 µg/L; Heparin 0–1000 units/L; Levocarnitine 263–2333 mg/L	Alprostadil, Pfizer	0.02 mg/L	Glucose 50 mg/mL	1 + 1	C/I	[10] C grade
	Ampicillin/sulbactam, AuroMedics	20 mg/mL	NaCl 9 mg/mL	1 + 1	C	
	Ampicillin, Athenex	30 mg/mL	NaCl 9 mg/mL	1 + 1	C	
	Bumetanide, Hospira; Westward	0.25 mg/mL	NA	1 + 1	C	
	Cisatracurium, AbbVie	2 mg/mL	NA	1 + 1	C/I	
	Dexmedetomidine, Baxter	0.04 mg/L	NA	1 + 1	C	
	Furosemide, Novaplus; Hospira	10 mg/mL	NA	1 + 1	C/I	
	Heparin, Fresenius Kabi	500 units/mL	NaCl 9 mg/mL	1 + 1	C/I	
	Hydromorphone, Teva	0.5 mg/mL	Glucose 50 mg/mL	1 + 1	C	
	Ketamine, Mylan	10 mg/mL	NA	1 + 1	C/I	
	Lacosamide, UCB	10 mg/mL	NA	1 + 1	C	
	Nicardipine, Westward	0.5 mg/mL	Glucose 50 mg/mL	1 + 1	C	
	Rocuronium, Sandoz	10 mg/mL	NA	1 + 1	C	
	Sildenafil, AuroMedics	0.8 mg/mL	NA	1 + 1	C/I	
	Vasopressin, Par Pharmaceutical	1 unit/mL	Glucose 50 mg/mL	1 + 1	C	
Six different PN solutions containing the following: Amino acids (Primene) 23–40 g/L; Glucose 75–120 g/L; Sodium 10–60 mmol/L; Potassium 0–22 mmol/L; Calcium 5–17 mmol/L; Magnesium 1.5 mmol/L; Phosphate 4–13 mmol/L; Acetate 0–34 mmol/L; Chloride 7.6–30.7 mmol/L; Zinc 0–3270 µg/L; Selenium 0–20 µg/L; Iodide 0–8.2 µg/L; Heparin 500 units/L	Pentoxifylline, Trental, Sanofi	5.0 mg/mL	NaCl 9 mg/mL	1 + 1	C	[11] D grade
PN solution containing the following: Amino acids (Travasol 10%) 30 g/L; Glucose 250 g/L; Sodium 150 mmol/L; Potassium 80 mmol/L; Calcium 9 mmol/L; Magnesium 2.5 mmol/L, Phosphate 7 mmol/L; Acetate 37.5 mmol/L; Chloride 75 mmol/L; Infuvite Pediatric 5 mL/L; Multitrace-4 1 mL/L; Selenium 10 µg/L; Heparin 1000 units/L	A. Epinephrine hydrochloride, Amphastar B. Milrinone lactate injection, Hikma West-Ward Pharmaceuticals C. Vasopressin, Par Pharmaceutical D. Calcium gluconate, Fresenius Kabi	A. 0.1 mg/L B. 1.0 mg/L C. 1 unit/mL D. 100 mg/mL	A. NaCl 9 mg/mL B. Undiluted C. Glucose 50 mg/mL D. Undiluted	1 + 1 + 1 + 1 + 1	C	[12] C grade
PN solution containing the following: Amino acids (TrophAmine) 5.7 g/L; Glucose 250 g/L; Sodium 23.1 mmol/L; Potassium 17.1 mmol/L; Calcium 2.9 mmol/L; Magnesium 1.4 mmol/L; Phosphate 3.9 mmol/L; Acetate 11.3 mmol/L; Multivitamin 5.7 mL/L; Trace elements 0.3 mL/L	Amiodarone hydrochloride, Bedford	16 mg/mL	Sterile water	1 + 1	I	[13] C grade
	Caffeine citrate, Ben Venue	20 mg/mL	Sterile water	1 + 1	C	
	Clindamycin phosphate, Hospira	24 mg/mL	Sterile water	1 + 1	C	
	Enalaprilat, Baxter	0.08 mg/mL	Sterile water		C	
	Epinephrine hydrochloride, Hospira	0.0096 mg/mL	Sterile water	1 + 1	C	
	Fluconazole, Baxter	2 mg/mL	Sterile water	1 + 1	C	
	Fosphenytoin sodium, Parke-Davis	50 mg/mL	Sterile water	1 + 1	C	
	Hydrocortisone sodium succinate, Pharmacia	25.6 mg/mL	Sterile water	1 + 1	C	
	Metoclopramide hydrochloride, Sicor	0.58 mg/mL	Sterile water	1 + 1	C	
	Midazolam hydrochloride, Baxter	0.48 mg/mL	Sterile water	1 + 1	C	
	Pentobarbital sodium, Abbott	48 mg/mL	Sterile water	1 + 1	I	
	Phenobarbital sodium, Baxter	64 mg/mL	Sterile water	1 + 1	I	
	Rifampicin, Bedford	0.3; 7.5; 15; 30 mg/mL	Sterile water	1 + 1	I	
Three different PN solutions containing the following: Amino acids (TrophAmine) 28.1–41.7 g/L; Glucose 100–150 g/L; Sodium 10.8–62.5 mmol/L; Potassium 20.1–72.9 mmol/L; Calcium 10.2–24.0 mmol/L; Magnesium 1.2–3.1 mmol/L; Phosphate 8.1–19.2 mmol/L; Pediatric multivitamin 16.1–41.7 mL/L; Pediatric trace elements 1.6–4.2 mL/L; Selenium 24.1–62.5 µg/L; Zinc 1607–4167 µg/L; Molybdenum 2.0–5.2 µg/L; Heparin 500 units/L; Cysteine 1.1–1.7 g/L; Ranitidine 8.0–41.7 mg/L; Levocarnitine 0–416.7 mg/L	Aminophylline solution, Hospira	2.5 mg/mL	glucose 50 mg/mL	1 + 1	C	[14] D grade
PN solution containing the following: Amino acids (TrophAmine) 37.5 g/L; Glucose 175 g/L; Sodium 25 mmol/L; Potassium 19 mmol/L; Calcium 9.5 mmol/L; Magnesium 1.9 mmol/L; Phosphate 4.2 mmol/L; Acetate 12.5 mmol/L; MVI- Ped 52 mL/L; Trace Element Injection 4, Pediatric 2.5 mL/L; Ranitidine 73 mg/L	Alprostadil, Bedford	0.015 mg/mL	Glucose 50 mg/mL; NaCl 9 mg/mL	1 + 1	C	[15] D grade
Three different PN solutions containing the following: Amino acids (Aminosyn, TrophAmine) 30–40 g/L; Glucose 200 g/L; Sodium 48–93 mmol/L; Potassium 40–60 mmol/L; Calcium 0.7–1.3 mmol/L; Magnesium 2.2–4 mmol/L; Phosphate 7.4–12.9 mmol/L; Multivitamin injection 5 mL/L; Pediatric trace elements 3 mL/L; L-cysteine 0–1.2 g/L; Heparin 500 units/L	Milrinone, Sanofi-Synthelabo	0.2 mg/mL	Glucose 50 mg/mL; NaCl 9 mg/mL	1 + 1	C	[16] D grade
Three different PN solutions containing the following: Amino acids (TrophAmine) 20–30 g/L; Glucose 100–200 g/L; Sodium 38–77 mmol/L; Potassium 20–40 mmol/L; Calcium 1.3 mmol/L; Magnesium 1.25 mmol/L; Phosphate 21.4 mmol/L; Acetate 14.5–29 mmol/L; Chloride 38–77 mmol/L; Multivitamin 20 mL/L; Trace elements 2 mL/L; L-cysteine 0.2–0.3 g/L; Heparin 0–500 units/L	Acetazolamide sodium, Lederle	100 mg/mL	According to SmPC	1 + 1	I	[17] D grade
	Acyclovir sodium, Burroughs Wellcome	7 mg/mL		1 + 1	I	
	Amikacin sulfate, Apothecon	5 mg/mL		1 + 1	C	
	Aminophylline, American Regent	5 mg/mL		1 + 1	I	
		25 mg/mL		1 + 1	I	
	Ampicillin sodium, Apothecon	100 mg/mL		1 + 1	I	
		250 mg/mL		1 + 1	I	
	Cefotaxime sodium, Hoechst-Roussel	60 mg/mL		1 + 1	C	
	Ceftazidime, Eli Lilly	60 mg/mL		1 + 1	C	
	Chlorothiazide sodium, Merck	28 mg/mL		1 + 1	I	
	Dexamethasone sodium phosphate, American Regent	4 mg/mL		1 + 1	C	
	Dobutamine, Eli Lilly	5 mg/mL		1 + 1	C	
	Dopamine, American Regent	3.2 mg/mL		1 + 1	C	
	Fentanyl, Elkins-Sinn	50 µg/mL		1 + 1	C	
	Furosemide, American Regent	10 mg/mL		1 + 1	C	
	Gentamicin sulfate, Elkins-Sinn	10 mg/mL		1 + 1	C	
	Metronidazole, Abbott	5 mg/mL		1 + 1	C	
	Morphine sulfate, Elkins-Sinn	1 mg/mL		1 + 1	C	
	Penicillin G potassium, Marsam	500.000 units/mL		1 + 1	C	
	Ranitidine hydrochloride, Glaxo	25 mg/mL		1 + 1	C	
	Tobramycin sulfate, Eli Lilly	10 mg/mL		1 + 1	C	
	Vancomycin hydrochloride, Eli Lilly	5 mg/mL		1 + 1	C	
	Zidovudine, Burroughs Wellcome	4 mg/mL		1 + 1	C	

SmPC—Summary of product characteristic. * To allow for the presentation of data found in scientific papers, some simplifications were made, and not all possible drug–PN combinations are presented as separate rows in the table. In most studies, combinations of medications with more than one PN admixture were tested, which differed in their composition (information in the first columns of the tables); hence, the results may include those for which drugs were compatible with select, but not all, PN admixtures, and such combinations were assigned the designation C/I. ** Evidence quality assessment: A grade—very high-quality studies, B grade—high-quality studies, C grade—medium-quality studies, D grade—low-quality studies.

**Table 2:** Compatibility studies of intravenous medications and 3-in-1 PN emulsions (with lipids).

Parenteral Nutrition	Drug Solution	Final Concentration of Drug	Diluent	Selected Mixing Ratio of Drug + PN	Compatibility/Incompatibility (C/I) *	Ref./Grade (A,B,C,D) **
Eight different PN admixtures containing the following: Amino acids (Aminoplasmal Paed/Primene) 19.8 g/L; Glucose 142.9 g/L; Lipids (Lipidem/ClinOleic/SMOFlipid/Intralipid) 39.7 g/L; Sodium 27.7 mmol/L; Potassium 19.9 mmol/L; Magnesium 2.0 mmol/L; Calcium 3.96 mmol/L; Phosphate 4.0 mmol/L; Cernevit 0.5 mL/L; Peditrace 1.0 mL/L	Ondansetron, Accord Healthcare	0.02 mg/mL	NaCl 9 mg/mL	1 + 1, 2 + 1	C	[18] C grade
	Hydrocortisone, Bausch Health Ireland Limited	0.98 mg/mL		1 + 1, 4 + 1	C	
	Dexamethasone, Bausch Health Ireland Limited	0.08 mg/mL		1 + 1, 2 + 1	C	
PN admixture: Numeta G13E; Calcium gluconate 11.7 mmol/L; Phosphate 8.3 mmol/L; Soluvit 10 vials/L; Vitlipid N Infant 100 mL/L; Peditrace 33.3 mL/L	Morphine hydrochloride, Orione	0.2 mg/mL	Glucose 50 mg/mL	1 + 1, 1 + 7, 1 + 39	C	[19] B grade
	Dopamine hydrochloride, Takeda	2 mg/mL	Undiluted	1 + 1, 1 + 6, 1 + 56	C	
	Cefotaxime, Villerton and MIP Pharma	40 mg/mL	Glucose 50 mg/mL	1 + 1, 9 + 1, 1 + 20	C	
PN admixture: Numeta G13E; Soluvit 10 vials/L; Vitlipid N Infant 100 mL/L; Peditrace 33.3 mL/L	Paracetamol, B. Braun	10 mg/mL	Undiluted	1 + 1, 1 + 10, 3 + 2	C	[20] B grade
	Vancomycin, MIP Pharma	5 mg/mL	Glucose 50 mg/mL	1 + 1, 1 + 2, 1 + 5	C	
	Fentanyl, Hameln	10 µg/mL	Glucose 50 mg/mL	1 + 1, 1 + 10, 1 + 20	C	
PN admixture containing the following: Amino acids (Vaminolact) 26.4–27.5 g/L; Glucose 54.2–56.4 g/L; Lipids (SMOFlipid) 23.6 g/L Sodium 16.0 mmol/L; Potassium 15.4–16.0 mmol/L; Calcium 4.5–4.6 mmol/L; Magnesium 1.9–2.0 mmol/L; Phosphate 8.0–10.3 mmol/L; Chloride 24.3–25.3 mmol/L; Sulfate 1.9–2.0 mmol/L; Soluvit 0–2.9 vials/L; Vitlipid N Infant 0–33.4 mL/L; Peditrace 8.0–14.5 mL/L	Ampicillin sodium, Bristol Myers Squibb	50 mg/mL	NaCl 9 mg/mL	1 + 10, 1 + 1, 2 + 1	C/I	[21] B grade
	Ceftazidime pentahydrate, Fresenius Kabi	40 mg/mL	Glucose 50 mg/mL	1 + 10, 1 + 1, 1 + 2	C/I	
	Fluconazole, B. Braun	2 mg/mL	Undiluted	1 + 10, 1 + 1, 9 + 1	C/I	
	Fosphenytoin sodium, Pfizer	10 mg/mL	Glucose 50 mg/mL	1 + 50, 1 + 1, 5 + 1	C/I	
	Furosemide, Nycomed; Takeda	2 mg/mL	NaCl 9 mg/mL	1 + 100, 1 + 1, 2 + 1	C/I	
	Metronidazole, B. Braun	5 mg/mL	Undiluted	1 + 10, 1 + 1, 5 + 1	C/I	
	Paracetamol, B. Braun; Fresenius Kabi	10 mg/mL	Undiluted	1 + 10, 1 + 1, 1 + 2	C/I	
Lipid emulsions: Intralipid; Nutrilipid; SMOFlipid	Alprostadil, Pfizer	20 µg/mL	Glucose 50 mg/mL	1 + 1	C/I	[22] C grade
	Ampicillin/sulbactam, West-Ward	20 mg/mL	Undiluted	1 + 1	C	
	Ampicillin, Sandoz	30 mg/mL	NaCl 9 mg/mL	1 + 1	C	
	Bumetanide, West-Ward	0.25 mg/mL	Undiluted	1 + 1	C	
	Cisatracurium, AbbVie	2 mg/mL	Undiluted	1 + 1	C/I	
	Dexmedetomidine, Baxter	0.004 mg/mL	Undiluted	1 + 1	C	
	Furosemide, Hospira	10 mg/mL	Undiluted	1 + 1	C	
	Gentamicin, Baxter	2 mg/mL	Undiluted	1 + 1	C/I	
	Heparin, Fresenius Kabi	500 units/mL	Undiluted	1 + 1	C	
	Hydromorphone, Hospira	2.5 mg/mL	Glucose 50 mg/mL	1 + 1	C	
	Ketamine, Mylan	10 mg/mL	Undiluted	1 + 1	C	
	Lacosamide, UCB	10 mg/mL	Undiluted	1 + 1	C	
	Levocarnitine, Leadiant	50 mg/mL	NaCl 9 mg/mL	1 + 1	C	
	Milrinone, Hospira	0.2 mg/mL	Undiluted	1 + 1	C	
	Ondansetron, Accord	2 mg/mL	Undiluted	1 + 1	C	
	Rocuronium, Hospira	10 mg/mL	Undiluted	1 + 1	C/I	
	Sildenafil, AuroMedics	0.8 mg/mL	Undiluted	1 + 1	C	
	Famotidine, APP Pharmaceuticals	2.5 mg/mL, 0.25 mg/mL	NaCl 9 mg/mL	1 + 1 1 + 1	C/I C	
Three different PN admixtures containing the following: Amino acids (Primene) 30.0–33.0 g/L; Glucose 75.0–100.0 g/L; Sodium 15–33 mmol/L; Potassium 0–1.0 mmol/L; Calcium 12.0 mmol/L; Magnesium 1.5 mmol/L; Phosphate 10.0 mmol/L; Acetate 5.0–40.0 mmol/L; Chloride 9.3–13.5 mmol/L; Zinc 0–326 µg/L; Selenium 0–2 µg/L; Iodide 0–0.8 µg/L; Heparin 500 units/L and Lipid emulsion (SMOFlipid); Soluvit N Infant 50 mL/L; Vitlipid N Infant 200 mL/L	Ibuprofen lysine, BOSC Sciences	1.25 mg/mL to 5 mg/mL	NaCl 9 mg/mL, RO water	1 + 1 mixtures of ibuprofen lysine and PN/glucose/IV SMOFlipid	C	[23] C grade
Two different PN admixtures: Olimel N5E; Soluvit 0–5.4 vials/L; Vitlipid N Adult 0–9.9 mL/L; Tracel 10 mL/L and Numeta G16E; Soluvit 0–5.4 vials/L; Vitlipid N Infant 0–57.1 mL/L; Peditrace 50 mL/L;	Ampicillin sodium, Bristol Myers Squibb; APP Pharmaceuticals; SAGENT	50 mg/mL	NaCl 9 mg/mL	Olimel: 1 + 1, 1 + 10, 2 + 1	I	[24] B grade
				Numeta: 1 + 1, 1 + 10, 2 + 1	I	
	Ceftazidime, Fresenius Kabi	40 mg/mL	Glucose 50 mg/mL	Olimel: 1 + 10, 1 + 1, 2 + 1	C	
				Numeta: 1 + 10, 1 + 1, 1 + 2	C	
	Clindamycin, Villerton; Pfizer; Stragen	10 mg/mL	Glucose 50 mg/mL	Olimel: 1 + 25, 1 + 1, 4 + 1	C	
				Numeta: 1 + 33, 1 + 1, 2 + 1	C	
	Dexamethasone, GALEN	0.5 mg/mL	Glucose 50 mg/mL	Olimel: 1 + 100, 1 + 1, 2 + 1	C	
				Numeta: 1 + 50, 1 + 1, 1 + 2	C	
	Fluconazole, B. Braun	2 mg/mL	Undiluted	Olimel: 1 + 5, 1 + 1, 4 + 1	C	
				Numeta: 1 + 10, 1 + 1, 11 + 1	C	
	Fosphenytoin sodium, Pfizer	10 mg/mL	Glucose 50 mg/mL	Olimel: 1 + 25, 1 + 1, 7 + 1	I	
				Numeta: 1 + 33, 1 + 1, 6 + 1	I	
	Furosemide, Nycomed; Takeda	2 mg/mL	NaCl 9 mg/mL	Olimel: 1 + 100, 1 + 1, 4 + 1	C	
				Numeta: 1 + 100, 1 + 1, 9 + 4	I	
	Metronidazole, B. Braun	5 mg/mL	Undiluted	Olimel: 1 + 5, 1 + 1, 3 + 1	C	
				Numeta: 1 + 8, 1 + 1, 6 + 1	C	
	Ondansetron hydrochloride, Copyfarm, Fresenius Kabi; Accord	0.2 mg/mL	Glucose 50 mg/mL	Olimel: 3 + 10, 1 + 1, 2 + 1	C	
				Numeta: 1 + 4, 1 + 1, 1 + 2	C	
	Paracetamol, Bristol Myers Squibb; Actavis	10 mg/mL	Undiluted	Olimel: 3 + 5, 1 + 1, 3 + 1	C	
				Numeta: 1 + 4, 1 + 1, 2 + 1	C	

* To allow for the presentation of data found in scientific papers, some simplifications were made and not all possible drug–PN combinations are presented as separate rows in the table. In most studies, combinations of medications with more than one PN admixture were tested, which differed in their composition (information in the first columns of the tables); hence, the results may include those for which drugs were compatible with select, but not all, PN admixtures and such combinations were assigned the designation C/I. ** Evidence quality assessment: A grade—very high-quality studies, B grade—high-quality studies, C grade—medium-quality studies, D grade—low-quality studies.

**Table 3:** Methods used for determining compatibility between medications and 2-in-1 PN solutions (without lipids).

Scope of Assessment	Method	Apparatus	References
Visible precipitation, color change, gas production	Visual assessment under white light and against a dark background	Not applied	[10,12,13,14,16,17,23]
		Fluorescent lighting (not specified)	[15]
	Polarized light viewer	Apollo I, Adelphi Manufacturing Co., Haywards Heath, Sussex, England	[11]
Tyndall beam effect	Light scattering	Intravenous Solution Visual/Clarity Inspection Station Contamination Control Laboratories, Livonia, MI, USA	[16,17]
		Fiber optic Tyndall beam (Schott KL 1600 LED, Germany) and red laser pen (630–650 nm, P 3010 RoHS, Chongqing, China)	[19,20,21,24]
Sub-visual particle counting	Light obscuration	AccuSizer 780 Optical Particle Sizer; Nicomp PSS, Santa Barbara, CA, USA	[19,20,21,24]
		HIAC 9703+, Beckman-Coulter, Indianapolis, IN, USA	[10]
	Backgrounded membrane imaging (BMI)	Horizon Subvisible Particle Analysis Instrument, Halo Labs, Burlingame, CA, USA	[10]
	Flow imaging microscopy	FlowCam VS, Yokogawa Fluid-Imaging Technologies Inc., Scarborough, ME, USA	[10]
Turbidity	Nephelometric	Model 2100N, Hach Company, Loveland, CO, USA	[13]
		2100Q Turbidimeter, Hach, Loveland, CO, USA	[12]
		TU52000 Laboratory Laser Turbidimeter (Hach Company, Loveland, CO, USA)	[18]
	Formazin nephelometric	2100Qis Turbidimeter, Hach Lange GmbH, Duesseldorf, Germany	[19,20,21,24]
pH	Potentiometry	Seven Compact, Mettler Toledo, Greifensee, Switzerland	[19,20]
		Metrohm 744 pH Meter, Metrohm AG, Herisau, Switzerland	[21,24]
		Ohaus Starter pH Meter, Parsippany, NJ, USA	[23]
Drug concentration	HPLC	Not specified	[11]

**Table 4:** Methods used for determining compatibility between medications and 3-in-1 PN emulsions (with lipids).

Scope of Assessment	Method	Apparatus	References
Visible precipitation, color change, gas production	Microscopy	Olympus BX51 Microscope, Olympus America, Melville, NY, USA	[23]
Particle size (mean droplet diameter, MDD)	Dynamic light scattering	Zetasizer Nano ZS, Malvern Instruments, Worcestershire, UK	[18,19,20,23]
	Laser diffraction	Mastersizer 2000 and Hydro 2000G Sample Dispersion Unit, Malvern Instruments, Worcestershire, UK	[21,24]
Particle size (percentage of fat residing in globules larger than 5 µm, PFAT5)	Light obscuration	AccuSizer 780 Optical Particle Sizer; Nicomp PSS, Santa Barbara, CA, USA	[19,20,21,24]
		HIAC 9703+ Liquid Particle Counting System, Beckman-Coulter Life Sciences	[22]
pH	Potentiometry	Seven Compact, Mettler Toledo, Greifensee, Switzerland	[18,19,20]
		Metrohm 744 pH Meter, Metrohm AG, Herisau, Switzerland	[21,24]
Osmolality	Freezing point depression	Osmometer 800CLG, TridentMed, Warsaw, Poland	[18]
Zeta potential	Electrophoretic light scattering	Zetasizer Nano ZS, Malvern Instruments, Worcestershire, UK	[18]
Drug concentration	HPLC	Shimadzu LC-20 AD Prominence Liquid Chromatogram connected to a DGU-20AS Prominence Degasser, SIL-20A HT Prominence Autosampler, and SPD-M2DA Prominence Diode Array Detector, Shimadzu Scientific Instruments, Kyoto, Japan	[23]

## Data Availability

The data presented in this study are available on request from the authors.
